# Depressive symptoms and cognitive deficits in a cancer patient submitted to chemotherapy with 5-Fluoracil: a case report

**DOI:** 10.1590/S1980-57642013DN70300013

**Published:** 2013

**Authors:** Sara Mota Borges Bottino, Célia Petrossi Gallo Garcia, Bernardo de Mattos Viana, Cássio Machado de Campos Bottino

**Affiliations:** 1 Department of Psychiatry, Federal University of São Paulo.; 2 Old Age Research Group, Institute of Psychiatry, University of São Paulo.

**Keywords:** cognitive deficits, dementia, cancer, chemotherapy, 5-Fluoracil

## Abstract

Cognitive deficits in cancer patients can be related to depression, anxiety, and the side effects of treatments such as fatigue. In this case report, we described an elderly patient with rectal adenocarcinoma, which presented depressive symptoms and memory complaints after treatment with 5-Fluoracil and Leucovorin. Depressive symptoms improved after two months but cognitive and functional impairment worsened suggesting the diagnosis of mild dementia. Structural and functional brain changes were seen on neuroimaging exams. Rivastigmine was introduced up to 12 mg/day, and after a one-year follow up the patient remained stable. Cognitive deficits can be a consequence of cancer therapies and a protocol to investigate deficits cognitive could be useful to the diferential diagnosis and management of elderly cancer patients submitted to chemotherapy.

## Abstract (pt)

Déficits cognitivos em pacientes com câncer podem estar relacionados à depressão, ansiedade e aos efeitos colaterais dos tratamentos, como fadiga. Neste relato de caso, descrevemos um paciente idoso com adenocarcinoma do reto, que apresentou sintomas depressivos e queixas de memória, após o tratamento com 5-Fluoracil e Leucovorin. Os sintomas depressivos melhoraram após dois meses, mas o comprometimento cognitivo e functional pioraram, sugerindo o diagnóstico de demência leve. Mudanças cerebrais estruturais e funcionais foram vistos em exames de neuroimagem. Rivastigmina foi introduzida até 12 mg/dia, e após um ano de seguimento o paciente permanecia estável. Déficits cognitivos podem ser consequência das terapias anti-neoplásicas e um protocolo para investigar déficits cognitivos pode ser útil para o diagnóstico diferencial e manejo de pacientes idosos com câncer submetidos à quimioterapia.

## INTRODUCTION

Cognitive changes in cancer patients were mation processing, and difficulties in concenassumed to be related to psychologi-tration, attention and memory which have cal factors such as depression or anxiety, or become collectively known as "chemobrain".^1^ other side effects of cancer treatments such Deficits in the same cognitive domains can as fatigue. Reports by patients undergoing be seen in depressive syndrome, which can chemotherapy have indicated that systemic also be a consequence of cancer therapies. chemotherapy can produce a wide range of Moreover depressive disorder is common in cognitive symptoms including slowed infor-patients with cancer, with prevalence rates ranging from 22% to 29%.^2^ These cognitive deficits are relevant for the differential diagnosis between depression and dementia, as well as between depression and normal aging. Age is a well-established risk factor for cognitive decline, and researchers have speculated that older patients may be more vulnerable to cognitive side effects of cancer treatments.^3,4^

The commonly used chemotherapy drug, 5-fluorouracil (5-FU) has been associated with chemobrain in reports by breast cancer survivors and shown to induce cognitive impairment and a reduction in hippocampal neurogenesis in a rat model of chemotherapy.^4,5^ As only a subgroup of cancer patients experience persistent posttreatment cognitive decline, the examination of risk factors for cognitive change and close monitoring of those at increased risk of converting to dementia in the future are important in the management and follow-up of these patients.^3,4^ In this case report, we describe an older patient with rectal adenocarcinoma, with memory complaints and depressive symptoms after treatment with 5-Fluoracil and Leucovorin.

## CASE REPORT

SF, male, age 66, widower, taxi driver, four years' schooling. Adenocarcinoma of the rectum (T3N1M0) was diagnosed in September 2008. Underwent neoadjuvant chemotherapy (2 cycles of 5-Fluoracil and Leucovorin) for five consecutive days and concurrent radiotherapy in addition to rectosigmoidectomy (Aug/2009). Adjuvant chemotherapy was performed with 3 cycles of modified FLOX (Oxaliplatin and Leucovorin). The daughter reported that after surgery, the patient developed memory deficits, mislaying things at home, exhibiting difficulties in supermarket shopping (needing to go back several times having forgotten to buy items), and in controlling his bank account. He got lost while driving his taxi and was recently involved in a car accident having jumped the red light. The daughter added that he had difficulties accepting the colostomy and sometimes forgot and/or refused to use the colostomy bag, but improved in the months subsequent to the surgery. History of social use of alcohol but after surgery had increased consumption, indulging in covert drinking during the chemotherapy. No signs or symptoms of tolerance or withdrawal.

**Figure 1:**
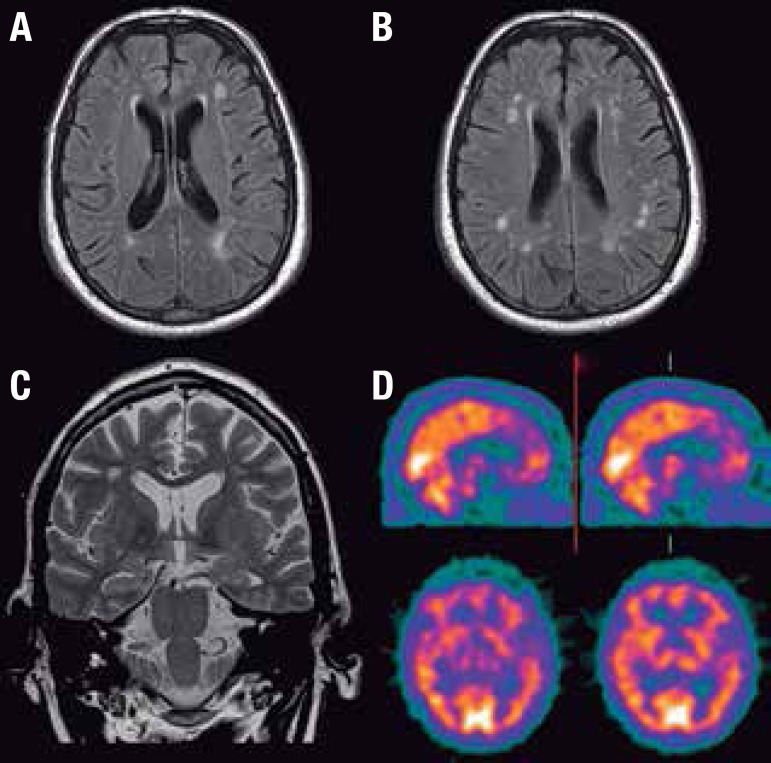
Flair MRI: Hyperintensity on deep white matter axial scans [A and B] and coronal images showing mild hippocampal atrophy [C]; Brain SPECT: hypoperfusion in right frontal lobe [D].

On psychiatric and mental status evaluation, he presented depressive mood, and symptoms including withdrawal, sadness and anedhonia. MMSE score was 25 and Bayer ADL scale score was 4.5, suggesting functional impairment. Severe temporal disorientation and deficits in verbal and visual episodic memory were seen on neuropsychological tests. Brain MRI showed structural changes in the central nervous system, including diffuse cortical atrophy and hyperintense lesions in deep white matter on axial scans (Figure 1A and 1B) and coronal images showing mild hippocampal atrophy (Figure 1C). Brain SPECT showed hypoperfusion in the right frontal lobe (Figure 1D).

Depressive symptoms improved (adjustment disorder) after two months but cognitive and functional impairment worsened suggesting a diagnosis of mild dementia. Rivastigmine was introduced up to 12 mg / day. After one-year follow up, the patient remained stable, with an MMSE=26, and Bayer ADL=4.96.

## DISCUSSION

The psychosocial distress and the symptoms of anxiety and depression presented by the cancer patients may result in difficulties for the diagnosis of cognitive dysfunction. Additionally, changes in attention, executive functions and slowed information processing, as seen in this case, are the most frequently described cognitive alterations in studies involving depressed patients, particularly those with late-onset depression, occurring after 60 or 65 years of age.^6^ However, persistent post-chemotherapy cognitive changes need to be examined within the broader context of risk factors and the biological processes associated with the cancer treatment and trajectory of normal aging.

In the case report, "the daughter added that he had difficulties accepting the colostomy and sometimes forgot and/or refused to use the colostomy bag, but improved in the months subsequent to the surgery" could be related to the adjustment disorder and also secondary to memory deficits after cancer therapies. On the follow-up, the patient presented severe temporal disorientation and deficits in verbal and visual episodic memory. Depressive symptoms improved (adjustment disorder) but cognitive and functional impairment worsened suggesting the diagnosis of mild dementia. The cognitive changes associated with chemotherapy are typically subtle (functioning is reduced but often remains within the normal range), and occur across various domains of cognition, including working memory, executive function and processing speed, but not the retrieval of remote memories. Furthermore, although acute cognitive changes during chemotherapy are common, long-term post-treatment cognitive changes seem to persist in only a subgroup (17-34%) of cancer survivors.^7^

Other psychiatric disorders must be ruled out as the primary cause of cognitive or functional impairment prior to determining a diagnosis of dementia syndrome. The main differential diagnoses include: depression, delirium, and use of psychoactive substances, including alcohol consumption. The patient's history of social use of alcohol, despite normal laboratory tests, and the increased consumption during chemotherapy, could have enhanced the toxic effects to the CNS of alcohol and 5-fluoracil.^4-6^ Batteries of neuropsychological tests can provide comprehensive evaluation of cognitive functioning, but their administration requires specialized training and can be time-intensive (1 to 6 hours), especially for patients dealing with cancer and its treatment. Several investigators have used shorter screening assessments of cognitive function, some of which include an overall score that can be used conveniently as an end point in clinical trials. Relatively brief measures include the High Sensitivity Cognitive Screen, the EXIT 25, a 25-item bedside measure of frontal function, and the CLOX, a clock-drawing task.^7^

Imaging studies demonstrated structural changes in the central nervous system, including hyperintense lesions in deep white matter on axial scans, coronal images showing mild hippocampal atrophy, and hypoperfusion in the right frontal lobe (Figure 1). Several cross-sectional, post-treatment studies utilizing magnetic resonance imaging (MRI) have documented reductions in gray matter, primarily in frontal structures and hippocampus, and white matter integrity in cancer survivors treated with chemotherapy, although negative results have also been reported^7^. Longitudinal studies have reported similar results: (i) decreased gray matter density in bilateral frontal, temporal (including hippo-campus), and cerebellar regions and right thalamus at 1 month post-chemotherapy with only partial recovery at 1 year post-chemotherapy in several structures, in contrast to no significant changes in gray matter over time in the no-chemotherapy cancer group and the healthy controls; and (ii) decreased frontal, parietal, and occipital white matter integrity in chemotherapy-exposed patients with no changes in either no-chemotherapy or healthy controls at post-treatment.^8,9^ Cross-sectional studies of cancer survivors utilizing functional imaging techniques, including functional MRI and functional positron emission tomography, have demonstrated areas of decreased activation during performance of a cognitive task in survivors exposed to chemotherapy, as compared with controls, in areas similar to sites of the structural differences described earlier.^10^ The patient presented abnormalities on MRI similar to those reported in these studies which could be associated to persistent cognitive impairment after treatment, as described in this case.

Previous studies have demonstrated brain changes after use of 5-Fluoracil at high doses. Baehring and Fulbright^11^ described a delayed leukoencephalopathy syndrome with distinct diffusion-weighted imaging abnormalities on MRI indicative of toxic white matter damage. This syndrome appeared to mimic a stroke-like syndrome and was seen mainly in patients receiving methotrexate, 5-fluorouracil (5-FU), carmofur, and capecitabine. First, the initial effect of cancer treatment may produce a cascade of biologic events, which causes continued cognitive decline with aging; and second, a given treatment may not be sufficient to cause enough redundancy loss to immediately affect cognitive function but may produce a delayed effect as aging continues. Support for each of these patterns was reported by Wefel et al.,^12^ who studied patients treated with regimens that included 5-FU: first, stable cognitive functioning over time after an acute posttreatment decline; second, continued cognitive decline over 1 year; and third, no acute cognitive decline with new evidence of cognitive decline at 1 year post-treatment. Despite the diagnosis of mild dementia, the patient remained stable at the 1 year follow-up. This evolution could be the result of the treatment with a cholinesterase inhibitor, reducing cognitive impairments associated to anti-cancer drugs, as suggested by Winocur et al.^13^ in a mouse model. However, further research is needed to confirm this initial finding.

In conclusion, the research so far suggests that performance changes in cognitive functioning can be seen in a subgroup of patients after chemotherapy, and that these changes might be associated with changes in brain structure and function. Therefore, it would be useful to perform cognitive and function evaluation in patients submitted to chemotherapy. A protocol to investigate such symptoms, as applied in the present case report, could be useful to aid the diagnosis and management of elderly cancer patients submitted to chemotherapy with drugs such as 5-FU.
